# PSAT1 is regulated by ATF4 and enhances cell proliferation via the GSK3β/β-catenin/cyclin D1 signaling pathway in ER-negative breast cancer

**DOI:** 10.1186/s13046-017-0648-4

**Published:** 2017-12-08

**Authors:** Song Gao, Anqi Ge, Shouping Xu, Zilong You, Shipeng Ning, Yashuang Zhao, Da Pang

**Affiliations:** 1 Department of Breast Surgery, Harbin Medical University Cancer Hospital, Harbin, Heilongjiang 150081 China; 2 Heilongjiang Academy of Medical Sciences, Harbin, Heilongjiang 150081 China; 3 Department of Epidemiology, Public Health College, Harbin Medical University, Harbin, Heilongjiang 150081 China

**Keywords:** PSAT1, Estrogen receptor (ER), Cell cycle, GSK3β/β-catenin/cyclin D1 pathway, ATF4

## Abstract

**Background:**

A growing amount of evidence has indicated that PSAT1 is an oncogene that plays an important role in cancer progression and metastasis. In this study, we explored the expression and function of PSAT1 in estrogen receptor (ER)-negative breast cancer.

**Method:**

The expression level of PSAT1 in breast cancer tissues and cells was analyzed using real-time-PCR (RT-PCR), TCGA datasets or immunohistochemistry (IHC). The overall survival of patients with ER-negative breast cancer stratified by the PSAT1 expression levels was evaluated using Kaplan-Meier analysis. The function of PSAT1 was analyzed using a series of in vitro assays. Moreover, a nude mouse model was used to evaluate the function of PSAT1 in vivo. qRT-PCR and western blot assays were used to evaluate gene and protein expression, respectively, in the indicated cells. In addition, we demonstrated that PSAT1 was activated by ATF4 by chromatin immunoprecipitation (ChIP) assays.

**Results:**

mRNA expression of PSAT1 was up-regulated in ER-negative breast cancer. A tissue microarray that included 297 specimens of ER-negative breast cancer was subjected to an immunohistochemistry assay, which demonstrated that PSAT1 was overexpressed and predicted a poor clinical outcome of patients with this disease. Our data showed that PSAT1 promoted cell proliferation and tumorigenesis in vitro and in vivo. We further found that PSAT1 induced up-regulation of cyclin D1 via the GSK3β/β-catenin pathway, which eventually led to the acceleration of cell cycle progression. Furthermore, ATF4 was also overexpressed in ER-negative breast cancers, and a positive correlation between the ATF4 and PSAT1 mRNA levels was observed in ER-negative breast cancers. We further demonstrated that knockdown of ATF4 by siRNA reduced PSAT1 expression. Finally, chromatin immunoprecipitation (ChIP) assays showed that PSAT1 was a target of ATF4.

**Conclusions:**

PSAT1, which is overexpressed in ER-negative breast cancers, is activated by ATF4 and promotes cell cycle progression via regulation of the GSK3β/β-catenin/cyclin D1 pathway.

**Electronic supplementary material:**

The online version of this article (doi: 10.1186/s13046-017-0648-4) contains supplementary material, which is available to authorized users.

## Background

Breast cancer is the most common female cancer and is the second leading cause of cancer-related deaths among females worldwide [1]. Estrogen receptor (ER)-positive breast cancers account for 60-70% of all breast cancers, but the remaining 30-40% of breast cancers are ER-negative breast tumors, which do not express ER, a protein with both prognostic and predictive value [2, 3]. Unfortunately, ER-negative breast cancers are resistant to endocrine therapy, which reduces recurrence and mortality rates whether chemotherapy is given or not [3, 4]. Therefore, ER-negative breast cancers recur and metastasize more readily, and consequently, patients with this cancer type have a worse prognosis and shorter survival rates compared with those with ER-positive breast cancers. This underscores the importance of the identification of new prognostic markers and additional drug targets for this class of breast cancer.

Serine plays an essential role in the synthesis of biomolecules that support cell proliferation. Recent evidence implies that hyperactivation of serine contributes to tumorigenesis [5]. Within cells, serine is synthesized through a three-step reaction. First, 3-phosphoglycerate is oxidized into phosphohydroxypyruvate (pPYR) by phosphoglycerate dehydrogenase (PHGDH). Successively, phosphohydroxypyruvate (pPYR) is catalyzed by phosphoserine aminotransferase (PSAT1) to produce phosphoserine (pSER), which is then dephosphorylated by 1-3-phosphoserine phosphatase (PSPH) to form serine. Two recent studies have reported that the gene encoding phosphoglycerate dehydrogenase (PHGDH) is amplified in a significant subset of human tumors, which supports the idea that metabolic reprogramming occurs as the result of genomic modifications of metabolic enzymes, which independently contribute to tumorigenesis [6, 7]. PSAT1 was found to be up-regulated in colon cancer, esophageal squamous cell carcinoma (ESCC) and non-small cell lung cancer (NSCLC), and has been shown to enhance cell proliferation, metastasis and chemoresistance, which all contribute to a poor prognosis [8–11]. However, the expression and underlying mechanism of PSAT1 in ER-negative breast cancer are not well understood. These observations have prompted us to speculate the role of PSAT1 in the initiation and development of ER-negative breast cancer.

Activating transcription factor 4 (ATF4) is a member of the cyclic adenosine monophosphate responsive element-binding (CREB) protein family, which has been reported to be a potent stress-response gene that is expressed in a wide variety of tumors [12, 13]. ATF4 can protect tumor cells against stresses and a range of cancer therapeutic agents via the regulation of cellular adaptation to adverse circumstances [14–19]. Previous studies have shown that ATF4 overexpression exists in many tumors, which suggests that it may play an important role in tumor formation, progression and metastasis [17, 19–22].

In the current study, PSAT1 was significantly up-regulated in ER-negative breast cancer and was correlated with a poor patient prognosis. Moreover, PSAT1 was found to be regulated by ATF4, which then activated the GSK-3β/β-catenin pathway. This resulted in the enhancement of cyclin D1 expression and the promotion of cell proliferation.

## Methods

### Patients and tissue specimens

The archival material used in this study was obtained from the Department of Pathology at the Harbin Medical University Cancer Hospital, and included tissues from 297 patients with histologically confirmed ER-negative breast cancer (Additional file 1) and 112 matched normal tissue samples from patients who presented from 2006 to 2007. For the extraction of protein and RNA, fresh tissues from individuals with ER-negative breast cancer and normal controls were collected and stored at −80 °C immediately after resection [23]. None of the patients received adjuvant chemotherapy, immunotherapy, or radiotherapy before surgery, and the patients with recurrent tumors, metastatic disease, bilateral tumors, or other previous tumors were excluded. Pathologists diagnostically examined tumors for confirmation of ER-negative breast cancer and benign breast diseases. After surgery, adjuvant systemic therapy was determined according to the National Comprehensive Cancer Network (NCCN) guidelines. This study was approved by the Ethical Committees of Harbin Medical University. Written informed consent was obtained from all subjects who participated in this study.

### Cell culture

MDA-MB-468, MDA-MB-231, MDA-MB-453, BT-549, HCC70, Hs578T and MCF-7 cells were cultured in RPMI-1640 or DMEM (Gibco, Carlsbad, CA, USA). All media were supplemented with 10% fetal bovine serum (FBS). MCF-10A cells were cultured as described previously [24]. All cells were incubated at 37 °C in humidified air containing 5% CO_2_.

### Plasmid, Lentivirus production and infection

Regarding the knock down of PSAT1, two human PSAT1 targeted RNAi (RNAi#1: TTCCAAGTTTGGTGTGATT; RNAi#2: ACTCAGTGTTGTTAGAGAT) sequences were obtained from GeneChem Co. Ltd. (Shanghai, China). As a control, scrambled versions of these sequences were used. The sequences shown above were inserted into the GV248 vector plasmid. For the overexpression of PSAT1, full-length human PSAT1 cDNA was cloned into the pLVX-puro vector. Lentiviral particles were constructed and packaged by Shanghai GeneChem Co. Ltd. Briefly, the cells were infected with lentivirus to generate stable cell lines. After 24 h, the cells were transferred to medium containing 4 μg/ml puromycin and were cultured for 3 days.

### Interfering RNA and transfection

ATF4 siRNAs and scrambled negative control siRNA were purchased from Invitrogen (Invitrogen, CA, USA). The siRNAs were transfected into cells using Lipofectamine 3000 (Invitrogen, CA) according to the manufacturer’s protocol. The sequences are as follows: ATF4-RNAi#1: 5′-CUGCUUACGUUGCCAUGAUTTAUCAUGGCAACGUAAGCAGTT-3′; ATF4-RNAi#2: 5′-CCCUUCAGAUAAUGAUAGUTTACUAUCAUUAUCUGAAGGGTT-3′; Scrambled-siRNA:5′-UUCUCCGAACGUGUCACGUTTACGUGACACGUUCGGAGAATT-3′.

### Cell proliferation assays

A cell proliferation assay was performed with a CCK-8 kit (Beyotime Institute of Biotechnology, Shanghai, China) according to the manufacturer’s instructions. Briefly, 2 × 10^3^ cells were plated in each well of a 96-well plates and were cultured overnight. According to the instructions, Cell Counting Kit-8 (CCK-8) reagent was added at 24, 48, 72 or 96 h and incubated at 37 °C for 1 h. Each assay was independently repeated three times in triplicate.

### Colony formation assays

Cells were plated into a 6-well plate and cultured in media containing 10% FBS for 14 days. Colonies were fixed in methanol for 30 min, and 500 μl 0.5% crystal violet was added (Sigma, St. Louis, MO, USA) to each well for 30 min for visualization and counting.

### Migration and invasion assays

Cells in serum-free media were placed into the upper chamber of an insert for the migration assays (8-μm pore size, Millipore), while for the invasion assays, the cells were seeded on plates coated with Matrigel (Sigma-Aldrich, USA). Medium containing 10% FBS was added to the lower chamber. After incubation at 37 °C for 12 h(Migration) or 24 h(Invasion), non-invading cells that remained in the top chambers were removed with a cotton swab, and the cells that had migrated to the underside of the membrane were fixed in 100% methanol for 30 min, air-dried, stained with 0.5% crystal violet, imaged, and counted under a light microscope.

### RNA preparation and qRT-PCR

Total RNA was extracted using TRIzol reagent according to the manufacturer’s protocol (Invitrogen, Beijing, China), and cDNA was synthesized using a PrimeScript RT reagent Kit with gDNA Eraser (Takara Bio, Otsu, Japan). mRNA expression was examined by real-time PCR using FastStart Universal SYBR Green Master (Roche, Mannheim, Germany) with gene-specific primers and an ABI 7500 Fast Real-time PCR Detection System (Applied Biosystems, Foster City, CA, USA). The results were normalized to the expression of β-actin. The sequences of the primers used were as follows: PSAT1-F: 5′-GTCCAGTGGAGCCCCAAAA-3′; PSAT1-R: 5′-TGCCTCCCACAGACCTATGC-3′; CCND1-F: 5′-GCTGCGAAGTGGAAACCATC-3′; CCND1-R: 5′-CCTCCTTCTGCACACATTTGAA-3′; β-actin-F: 5′-CAACCGCGAGAAGATGACC-3′; β-actin-R: 5′-ATCACGATGCCAGTGGTACG-3′.

### Flow cytometry analysis

Cells were seeded in 6-well plates, and after 24 h, the cells were harvested and washed twice with cold PBS. For the cell cycle analysis, the cells were fixed in ice-cold 75% ethanol overnight at 4 °C. After fixation, the cells were washed and resuspended twice in PBS and were then incubated with propidium iodide (BD Bioscience, San Jose, CA, USA) and RNase for 30 min at room temperature. For the cell apoptosis analysis, the cells were stained with PE Annexin V and 7-AAD (BD Bioscience, San Jose, CA, USA) for 15 min at room temperature. The cells were then analyzed using a FACSCalibur flow cytometer (BD Biosciences, San Jose, CA, USA).

### Western blotting analysis

Cultured cells or frozen tissue samples were harvested and lysed in RIPA buffer consisting of a 1% protease inhibitor mixture. A western blotting assay was performed as previously described [25]. The following antibodies were used: anti-PSAT1(Abcam, Cambridge, MA, USA, 1:1000), anti-Cyclin D1 (Abcam, Cambridge, MA, USA, 1:1000), anti-p-GSK-3β (Cell Signaling Technology, Beverly, MA, USA, 1:1000), anti-GSK-3β (Wanleibio, Shenyang, China,1:500), anti-β-catenin (Wanleibio, Shenyang, China, 1:500), and anti-ATF4 (Cell Signaling Technology, Beverly, MA, USA, 1:1000); anti-β-Tubulin (Santa Cruz Biotechnology, CA, USA, 1:1000) as an internal control.

### Immunohistochemistry (IHC)

A tissue microarray (TMA) that included samples from 297 consecutive patients with histologically confirmed estrogen receptor-negative breast cancer and 112 controls was generated according to a previously described method [26]. The tissue sections were dried at 70 °C for 3 h for deparaffinization and hydration. Subsequently, the sections were washed with phosphate-buffered saline (PBS; 3 × 3 min). The washed sections were treated with 3% H_2_O_2_ in the dark for 5 to 20 min. After washing in distilled water, the sections were again washed with PBS (3 × 5 min). Antigen retrieval was performed in citrate buffer (pH 6.0) at 100 °C for 10 min. Each section was incubated with the polyclonal primary rabbit antibody against PSAT1 at a 1:100 dilutions (Abcam, Cambridge, MA, USA) overnight at 4 °C. After washing with PBS (3 × 5 min), each section was further incubated with an anti-rabbit secondary antibody (1:200; Abcam, Cambridge, MA, USA) at room temperature for 30 min. After another wash in PBS (3 × 5 min), each section was immersed in 500 μl of diaminobenzidine (DAB) working solution at room temperature for 3 to 10 min. Finally, the slides were counterstained with hematoxylin and mounted in crystal mount medium. PSAT1 expression was analyzed and scored independently by two observers based on the intensity and the distribution of positively stained tumor cells, which were demarcated by yellow particles observed in the cytoplasm. The PSAT1 staining index was classified into four groups: level 0 (no staining), level 1 (0-20% of tumor cells stained), level 2 (20-50% of tumor cells stained) and level 3 (>50% of tumor cells stained). Overall expression was then graded as either negative expression (level 0) or positive expression (levels 1-3) [8].

### Animal experiments

Animal experiments were approved by the Medical Experimental Animal Care Commission of Harbin Medical University. BALB/C-nu/nu nude mice were obtained from Beijing Vital River Laboratory Animal Technology Company. Approximately 5 × 10^6^ cells (HCC70-NC or HCC70-KD) or 8 × 10^6^ cells (BT-549-Vector or BT-549-PSAT1) in 200 μl of serum-free medium were injected directly into the right dorsal flank per mouse. Tumor growth was measured with calipers every 3 days, and the tumor volumes were calculated using the formula: 1/2 (length × width^2^). Mice were euthanized and tumor weight was examined 27 days after the injections.

### Chromatin immunoprecipitation assay

Chromatin immunoprecipitation (ChIP) assays were performed using the ChIP Assay Kit (Beyotime, Shanghai, China) according to the manufacturer’s protocol with slight modifications. Cells were cross-linked with 1% formaldehyde and terminated after 10 min by the addition of glycine at a final concentration of 0.125 M. DNA was immunoprecipitated from the sonicated cell lysates using an ATF4 antibody (Cell Signaling Technology, Beverly, MA, USA); IgG (BD Biosciences, San Diego, CA, USA) served as the negative control. The DNA was subjected to PCR to amplify the ATF4 binding sites. The amplified fragments were then analyzed on an agarose gel. Chromatin (10%) was used before immunoprecipitation as the input control. The primer sequence was as follow: 5′-GTTTGCATCCCTGCGTGT-3′ and 5′-CCGAGCTTCCTCACCAACT-3′.

### Statistical analyses

Data analyses were performed using Graph Pad (GraphPad Prism, La Jolla, CA, USA), Excel (Microsoft Corp, Redmond, WA, USA) and SPSS 20.0 (SPSS, Chicago, IL, USA. The Chi-square test was used to assess correlations between PSAT1 expression and the clinicopathological features of ER-negative breast cancer patients. Survival curves were generated using the Kaplan-Meier method and the log-rank test. Student’s t-test was used to determine significant differences between two experimental conditions. Data from The Cancer Genome Atlas for breast invasive carcinoma (TCGA BRCA) were downloaded from the UCSC Xena Database (https://tcga.xenahubs.net/download/TCGA.BRCA.sampleMap/HiSeqV2.gz; Full metadata) and were used to detect PSAT1 and ATF4 expression in various types of breast cancer. The level of significance was set at *P* < 0.05.

## Results

### PSAT1 was overexpressed in ER-negative breast cancer specimens as well as in breast cancer cell lines

**Fig. 1:**
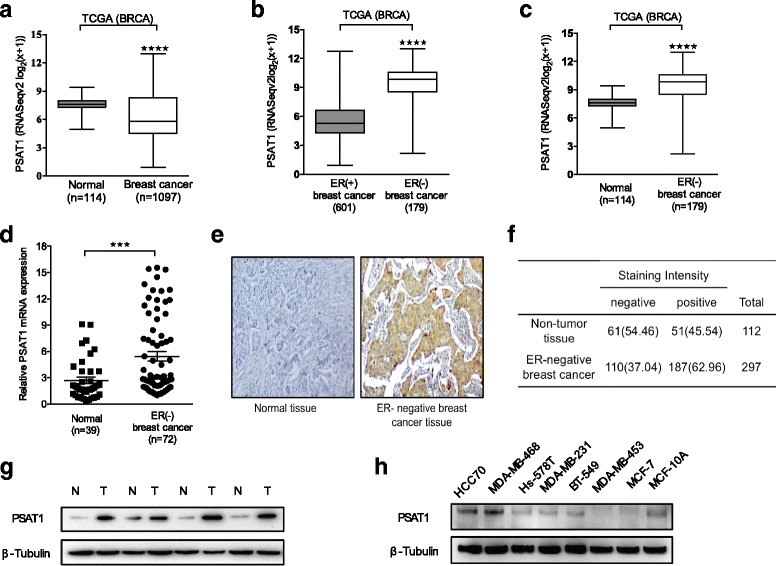
PSAT1 was overexpressed in ER-negative breast cancer specimens and cell lines. **a** Expression profile of PSAT1 in primary breast cancer tissues (*n* = 1097) and normal breast tissues (*n* = 114) (TCGA). **b** PSAT1 expression levels were higher in ER-negative breast cancer compared with ER-positive breast cancer (TCGA). **c** Expression of PSAT1 in 170 ER-negative breast cancers and 114 normal breast tissues (TCGA). **d** Relative expression of PSAT1 in ER-negative breast cancers (*n* = 72) in comparison with non-tumor normal tissues (*n* = 39). Data are presented as the fold-change in ER-negative breast cancers relative to normal tissues. **e** Representative images of PSAT1 immunohistochemical staining. **f** Table reports the numbers of ER-negative breast cancers versus non-tumor tissues with ‘positive’ or ‘negative’ PSAT1 staining. (Chi-square test is used. *P* < 0.01). **g** Western blotting analysis of PSAT1 expression in ER-negative breast cancers and paired normal tissues. The levels of β-Tubulin were used as an internal control. T: ER-negative breast cancer N: normal tissue**. h** Western blotting for PSAT1 in 7 human breast cancer cell lines and non-transformed MCF-10A. ***P < 0.001, ****P < 0.0001. TCGA = The Cancer Genome Atlas; BRCA = Breast carcinoma

To investigate the potential role of PSAT1 in breast cancer, we first analyzed PSAT1 mRNA expression in breast cancer RNAseq data from the TCGA (Fig. 1a–c). We found that PSAT1 expression was significantly down-regulated in breast cancers compared with normal tissues. Interestingly, PSAT1 expression was dramatically up-regulated in ER-negative breast cancers compared with ER-positive BC and normal tissues (*P* < 0.0001). Moreover, the results of the TCGA data analysis were validated using real-time PCR in 72 ER-negative breast tumors and 39 non-cancerous breast tissues (Fig. 1d). These results confirmed that the expression levels of PSAT1 mRNA were significantly increased in ER-negative breast cancers compared with non-tumor tissues. Next, the difference in PSAT1 protein expression levels between ER-negative BC and normal breast tissues was investigated using immunohistochemistry and western blotting. By analyzing 297 ER-negative breast tumor samples and 112 non-cancerous samples by immunohistochemistry for PSAT1, positive staining (brown) was detected in the majority of ER-negative tumor tissues but was detected less frequently in non-cancerous tissues (Fig. 1e, f), indicating that protein expression of PSAT1 is elevated in ER-negative breast cancers compared with non-tumor tissues (*p* = 0.002). The same trend was also observed in the western blotting analysis (Fig. 1g). Across a set of breast cancer cell lines, ER-negative breast cancer cell lines had higher PSAT1 protein expression compared with non-transformed MCF-10A and MCF-7 (ER-positive breast cancer cell lines) (Fig. 1h). This is consistent with the finding that PSAT1 expression was up-regulated at the mRNA and protein levels in a higher fraction of ER-negative breast cancers. Overall, ER-negative breast cancer cell lines and tissues exhibited relatively higher levels of PSAT1 expression. These results imply that PSAT1 overexpression may play an important role in the development of ER-negative breast cancer.

### The clinical significance of PSAT1 in patients with ER-negative breast cancer

**Fig. 2:**
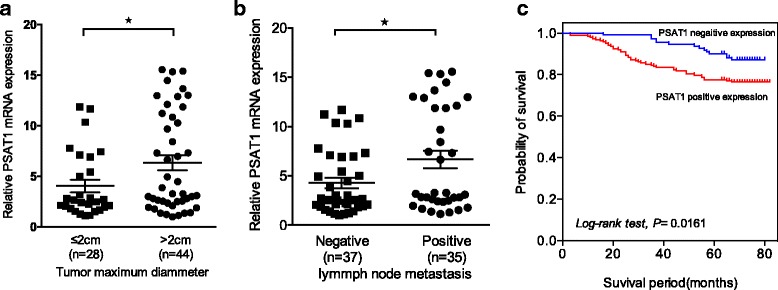
The clinical significance of PSAT1 overexpression in ER-negative breast cancer patients. The expression levels of PSAT1 in ER-negative breast cancers were divided according to tumor size (**a**) and the number of lymph node metastases (**b**). (*P < 0.05) (**c**) Kaplan-Meier survival analyses show that high expression of PSAT1 is correlated with poor survival (*n* = 297; *P < 0.05, log-rank test)

To further investigate whether PSAT1 overexpression is involved in ER-negative breast cancer progression, the correlation between PSAT1 levels and the clinicopathological features of ER-negative breast cancers was examined. The relative mRNA expression levels of PSAT1 were analyzed in 72 ER-negative breast tumors, and PSAT1 up-regulation was strongly associated with tumor size (*P* < 0.05, Fig. 2a) and axillary lymph node metastasis (*P* < 0.05, Fig. 2b). As shown in Table 1, statistical analyses using IHC results revealed that PSAT1 was positively correlated with tumor size (*P* = 0.024), TNM stage (*P* = 0.026) and Ki67 status (*P* < 0.028). We also detected PSAT1 expression in 107 of the 145 (73.79%) patients with histological grade I and II tumors, and in 80 of 152 (52.63%) patients with grade III tumors (*P* < 0.001). However, no significant association was found between PSAT1 and age, LNM, Her-2 status or P53 status. Therefore, we hypothesize that high expression of PSAT1 may be involved in tumor cell proliferation and may play an important role in ER-negative breast cancer development. Additionally, the Kaplan-Meier 5-year survival analysis showed that patients with ER-negative breast cancer with higher expression of PSAT1 had a remarkably poorer prognosis than those with low PSAT1 expression (*P* = 0.016, log- rank test; Fig. 2c). Together, high expression of PSAT1 may serve as a biomarker for poor prognosis in ER-negative breast cancer.

**Table 1:** Correlation between PSAT1 expression and the clinicopathological features of ER-negative breast cancers

Characteristics	NO	PSAT1 expression		*P-*value
	(*n* = 297)	Negative	Positive	
Age				
< 50	165	64 (38.79)	101 (61.21)	0.546
≥ 50	132	46 (34.85)	86 (65.15)	
Tumor size				
≤ 2 cm	105	48 (45.71)	57 (54.29)	0.024
> 2 cm	192	62 (32.29)	130 (67.71)	
LNM				
Negative	144	55 (38.19)	89 (61.81)	0.719
Positive	153	55 (35.95)	98 (64.05)	
TNM stage				
I	62	31 (50.00)	31 (50.00)	0.026
II;III	235	79 (33.62)	156 (66.38)	
Histological grade				
I;II	145	38 (26.21)	107 (73.79)	<0.001
III	152	72 (47.37)	80 (52.63)	
Her-2 status				
Negative	239	92 (38.49)	147 (61.51)	0.363
Positive	58	18 (31.03)	40 (68.97)	
Ki67 status				
Negative	111	63 (56.76)	48 (43.24)	<0.001
Positive	186	47 (25.27)	139 (74.73)	
P53 status				
Negative	95	33 (34.74)	62 (65.26)	0.608
Positive	202	77 (38.12)	125 (61.88)	

*LNM* lymph node metastasis

### Manipulation of PSAT1 levels in ER-negative breast cancer cells

We performed western blotting analysis to compare the expression levels of PSAT1 in various ER-negative breast cancer cell lines and non-transformed MCF-10A and ER-positive MCF-7-cell lines. As shown in Fig. 1h, HCC-70 and MDA-MB-468 cells expressed higher levels of PSAT1 than the other cell lines. Therefore, to determine the function of PSAT1 in ER-negative breast cancer cells, HCC70 and MDA-MB-468 cells were infected with two specific short hairpin RNAs (shRNAs) using a lentivirus-mediated system to generate HCC70-KD and MDA-MB-468-KD cell lines. BT-549 stable PSAT1-overexpressing cells were established with a PSAT1-vector using a lentivirus-mediated system.

**Fig. 3:**
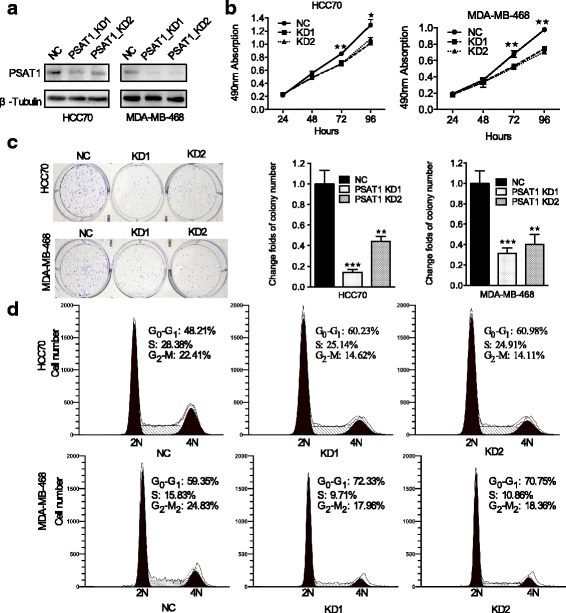
Knockdown of PSAT1 inhibited tumorigenicity of ER-negative breast cancer cells. **a** Western blot shows PSAT1 expression in HCC70 and MDA-MB-468 cells infected with Lenti-shPSAT1 or control. β-tubulin was used as a loading control. **b** CCK-8 assay was performed to determine the effect of PSAT1 silencing on the proliferation of the indicated cells at the indicated time points. **c** Knockdown of PSAT1 suppressed the colony formation ability of HCC70 and MDA-MB-468 cells compared with that of control cells. The values of the control cells were normalized to 1. For (**b**) and (**c**), the results are expressed as the mean ± SD; *n* = 3. **d** Cell cycle analysis of the indicated cells according to flow cytometry. *p < 0.05. **p < 0.01, ***P < 0.001

**Fig. 4:**
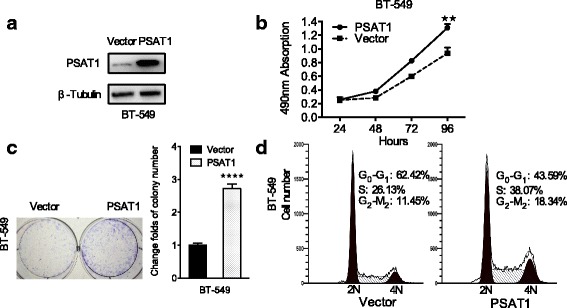
Overexpression of PSAT1 promoted the proliferation of ER-negative breast cancer cells. **a** Overexpression of PSAT1 in BT-549 cells was analyzed by WB. β-tubulin was used as a loading control. **b** The proliferation of BT-549 cells with stably up-regulated PSAT1 were tested by CCK-8 assay. **c** Overexpression of PSAT1 enhanced the colony formation ability of BT-549 cells. The values of the vector-control cells were normalized to 1. In (B) and (C), the results are expressed as the mean ± SD; *n* = 3. **d** The cell cycle was analyzed in BT-549 cells with stable overexpression of PSAT1 by flow cytometry. **p < 0.01. ****p < 0.0001

Then, we detected the protein expression level of PSAT1 in these target cells. As shown in Figs. 3a and 4a, compared with control cells, PSAT1 was significantly knocked down in MDA-MB-468-KD and HCC70-KD cells, but PSAT1 expression was increased in BT-549-PSAT1 cells.

### Knockdown of PSAT1 inhibited tumorigenicity of ER-negative breast cancer cells

To investigate the potential role of PSAT1 in ER-negative breast cancer cells, CCK-8 assays were performed in HCC70 and MDA-MB-468 cells to measure the cell viability. As shown in Fig. 3b, the knockdown of PSAT1 significantly suppressed the viability of these two breast cancer cell lines compared with control cells. Moreover, the colony formation ability of these cells was drastically inhibited after PSAT1 was silenced compared with their respective controls (Fig. 3c).

Given that the knockdown of PSAT1 inhibited the proliferation of ER-negative breast cancer cells, we sought to explore the underlying mechanisms using flow cytometry analysis. As shown in Fig. 3d, the flow cytometry results supported the idea that the suppression of PSAT1 led to a remarkable increase in the proportion of cells in G0/G1 phase, as well as a notable decrease in the proportion of cells in S phase compared with negative control HCC70 and MDA-MB-468 cells. Taken together, these results indicate that the knockdown of endogenous PSAT1 suppressed cell proliferation in vitro and inhibited G1/S transition of ER-negative breast cancer cells.

### Overexpression of PSAT1 promoted breast cancer cell proliferation in vitro

To further validate the role of PSAT1 in the proliferation of ER-negative breast cancer cells, exogenous PSAT1 was stably transduced into BT-549 cells (Fig. 4a). As expected, compared with control cells, ectopic overexpression of PSAT1 significantly increased proliferation (Fig. 4b). Similarly, the result of the colony-formation assay showed that clonogenic survival was enhanced following elevated PSAT1 expression in BT-549 cells (Fig. 4c). As shown in Fig. 4d, flow cytometry showed that ectopic PSAT1 expression markedly increased the proportion of S-phase cells and decreased the percentage of cells in G0/G1 phase. Collectively, these results suggest that exogenous PSAT1 promoted G1/S transition and thus enhanced the proliferation of ER-negative breast cancer cells.

### PSAT1 enhanced tumor formation of ER-negative breast cancer cells in a xenograft model

**Fig. 5:**
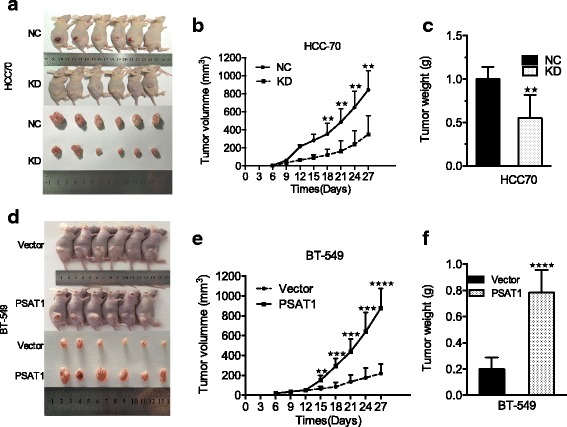
PSAT1 promoted tumor formation in a nude mouse model. **a** PSAT1-knockdown or control HCC70 cells and (**d**) PSAT1-overexpression or vector BT-549 cells were injected into the flank of nude mice respectively (upper panel). Tumors were resected from mice 27 days (lower panel). **b** and **e** Volume of indicated tumor were shown using time-course line plot. Tumor volume was calculated every 3 days. **c** and **f** Tumor weight of xenograft in indicated mice. Data are presented as the mean ± SD. (*n* = 6). **p < 0.01, ***P < 0.001, ****p < 0.0001

Immunodeficient BALB/c mice carrying HCC70 and HCC70-KD1 tumor cells were used to ascertain the role of PSAT1 in the tumorigenesis of ER-negative breast cancer in vivo. HCC70-NC and HCC70-KD1 tumor cells were delivered subcutaneously into nude mice, and after 27 days of growth, the tumors were harvested and analyzed (Fig. 5a). As expected, the silencing of PSAT1 significantly suppressed HCC70 tumor growth in mice compared with the control group. The mean tumor volume (Fig. 5b) was significantly decreased from 844.0 ± 87.31 mm^3^ to 350.7 ± 83.69 mm^3^ and the mean tumor weight (Fig. 5c) declined from 1.000 ± 0.05774 g to 0.5500 ± 0.1088 g (both *p* < 0.001). Moreover, we also performed xenograft studies using BT-549 stably overexpressed for PSAT1(Fig. 5d). As shown in the Fig. 5e and f, the in vivo tumor volume and weight of BT-549 cells was significantly increased from 218.3 ± 40.28 mm^3^ to 877.0 ± 81.04 mm^3^ (*p* < 0.0001) and from 0.2000 ± 0.03651 g to 0.7833 ± 0.07032 g (*p* < 0.0001) respectively, when PSAT1 was overexpressed. These results suggest that PSAT1 enhanced tumor growth of ER-negative breast cancer in vivo.

### PSAT1 regulated the expression of cyclin D1 through the GSK3β/β-catenin pathway

**Fig. 6:**
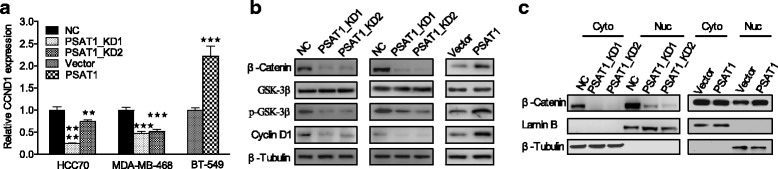
PSAT1 regulated Cyclin D1 expression through GSK3β/β-catenin. **a** The levels of cyclin D1 mRNA were analyzed by qRT-PCR in the indicated cells. The values of the NC and Vector groups were normalized to 1. **b** The protein expression levels of β-catenin, total GSK-3β, p-GSK-3β (Ser9) and cyclin D1 were examined by western blotting in the indicated cells. **c** Western blotting was performed in conjunction with cell fractionation to analyze the nuclear translocation and expression of β-catenin in the indicated cells. **P < 0.01, ***P < 0.001, ****P < 0.0001

Next, we investigated a potential mechanism for PSAT1 in the promotion of ER-negative breast cancer cell proliferation. Considering the function of PSAT1 in the promotion of G1/S phase transition in ER-negative breast cancer cells, cyclin D1, which is well known as an important regulator of G1 to S phase progression in many different cell types, was assessed by western blotting. As predicted, the expression of cyclin D1 was decreased in PSAT1-suppressed HCC70 and MDA-MB-468 cells but was increased in PSAT1-overexpressing BT-549 cells (Fig. 6a). Glycogen Synthase Kinase-3β (GSK3β), a serine/threonine protein kinase, has been considered as a potential tumor suppressor due to its ability to phosphorylate other proteins; it also has numerous cellular targets including cyclin D1 and β-catenin [27, 28]. Hence, we detected the expression of GSK3β and determined its phosphorylation status. As shown in Fig. 6a, the phosphorylation of GSK3β was inhibited when PSAT1 was silenced but was enhanced by the introduction of ectopic PSAT1. Interestingly, the PSAT1 expression level was positively correlated with β-catenin expression (Fig. 6a and b). In addition, we also observed that the up-regulation of PSAT1 expression markedly promoted the accumulation of cytoplasmic/nuclear β-catenin and caused the translocation of β-catenin from the cytoplasm to the nucleus. In contrast, the inhibition of PSAT1 dramatically attenuated the protein level of β-catenin in both the cytoplasm and the nucleus (Fig. 6c).

### PSAT1 is an important target of ATF4

**Fig. 7:**
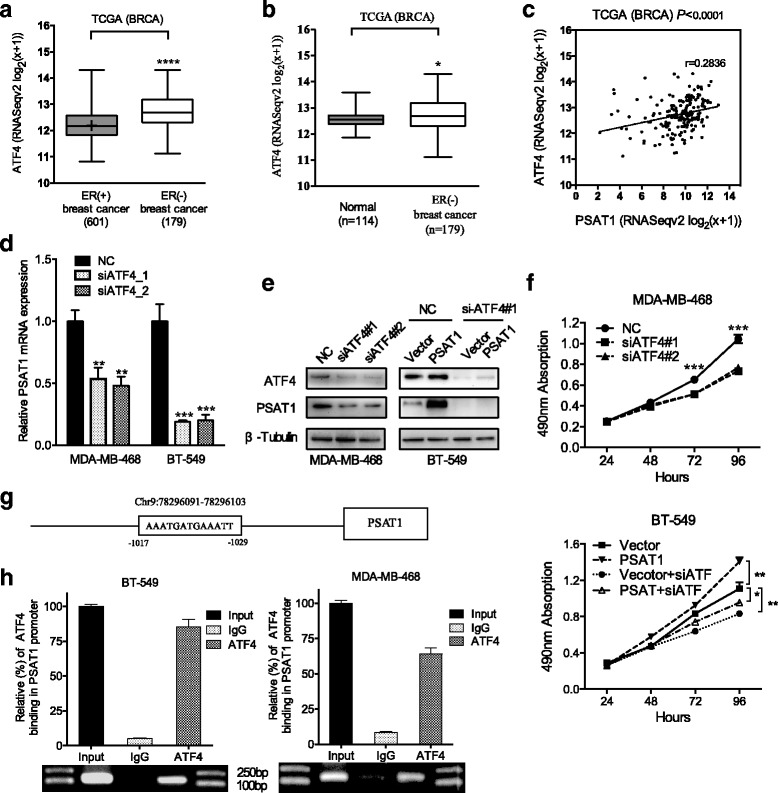
PSAT1 is a target of ATF4 in ER-negative breast cancer. **a** Expression profile of ATF4 in ER-negative breast cancer tissues (*n* = 179) and ER-positive breast cancer tissues (*n* = 601) (TCGA). **b** Relative expression of ATF4 in ER-negative breast cancer tissues (*n* = 179) in comparison with non-tumor normal tissues (*n* = 114) (TCGA). **c** Correlation between PSAT1 and ATF4 mRNA expression in samples from 179 patients with ER-negative breast cancer from the TCGA database. **d** Real-time PCR analysis show ATF4 siRNA inhibited the mRNA expression of PSAT1 in MDA-MB-468 and BT-549 cells compared to negative control cells. . These results were normalized to the expression of β-actin. The values of the NC group were normalized to 1. **e** Western blot shows PSAT1 expression was decreased in ATF4 silenced MDA-MB-468 and BT-549 cells compared to negative control cells. **f** The growth rate of the indicated cells was evaluated using a CCK-8 assay. **g** Schematic representation of the predicated ATF4 binding site within the PSAT1 promoter. **h** RT-PCR of the ChIP products validated the binding capacity of ATF4 to the PSAT1 promtor. The results of IgG were normalized to 1. *P < 0.05, **P < 0.01, ***P < 0.001, ****P < 0.0001. TCGA = The Cancer Genome Atlas; BRCA = Breast carcinoma

A previous study has shown that ATF4 transcriptionally activates serine biosynthetic genes in response to serine starvation in non-small cell lung cancer cells [29]. We found that the expression of ATF4 was significantly up-regulated in ER-negative breast cancers compared with ER-positive breast cancers and normal tissue (Fig. 7a and b). As shown in Fig. 7c, a positive correlation was observed between PSAT1 and ATF4 in ER-negative breast cancers (*r* = 0.2836; *P* < 0.0001). Consistently, in our current study, the silencing of ATF4 significantly reduced the mRNA and protein expression of PSAT1 (Fig. 7d and e). CCK-8 assays revealed that the silencing of ATF4 significantly reduced cell growth compared with the control (Fig. 7f top). Moreover, cell growth activity enhanced by PSAT1 required ATF4 because the knockdown of ATF4 not only decreased the proliferation rate but also attenuated the enhanced effect caused by stable overexpression of PSAT1 (Fig. 7f, bottom). To further validate our results, promoter scanning using the JASPAR Database showed that the PSAT1 promoter region contains a highly likely ATF4 binding site (Fig. 7h). ChIP assays further confirmed that ATF4 was efficiently bound to the PSAT1 promoter-specific region in both BT-549 and MDA-MB-468 cells. Taken together, these data indicate that ATF4 directly enhanced PSAT1 expression in ER-negative breast cancer.

## Discussion

It is well known that cancer cells possess distinct metabolic characteristics that are distinguishable from those of nonmalignant cells. Recent evidence has shown that serine metabolic reprogramming is due to the corresponding genetic changes in metabolic enzymes and that these gene modifications independently contribute to tumorigenesis [6, 7].

PSAT1 is the protein-coding gene of phosphoserine aminotransferase, which catalyzes serine biosynthesis. PSAT1 is overexpressed in colon cancers where it contributes to cell proliferation and chemoresistance, which result in a poor prognosis [9]. Liu et al. [8] have shown that PSAT1 expression was elevated in ESCC and that it was significantly associated with disease stage, lymph node metastasis, distant metastasis and poor outcome. A recent study has shown that the expression of PSAT1 was up-regulated in NSCLC, which was verified by an IHC assessment of 138 specimens and a qRT-PCR assay and its overexpression has also been associated with a poor prognosis of NSCLC. Martens et al. [30, 31] showed that PSAT1 inactivation by promoter methylation and low mRNA levels were both associated with a good outcome after tamoxifen treatment in ER-positive breast cancer. Our current study found for the first time that the expression of PSAT1 was significantly up-regulated in ER-negative breast cancers compared with ER-positive breast cancers, which was supported by the TCGA dataset. We then confirmed this finding by IHC using a tissue microarray, qRT-PCR and western blotting. Statistical analysis of these results showed that PSAT1 up-regulation was correlated with tumor development and poor prognosis.

Previous studies have shown that PSAT1 plays a vital role in cell proliferation as it acts as an oncogene in colon cancer and NSCLC [9, 11]. Possemato et al. [6] have illustrated that, through the suppression rate of the serine product, the inhibition of PSAT1 significantly decreased the proliferation of ER-negative breast cancer cells (MDA-MB-468 and BT-20) but not ER-positive breast cancer cells (MCF7). In this study, we also identified the function of PSAT1 in ER-negative breast cancer cells by applying gain- and loss-of-function approaches. We found that PSAT1 regulates the expression of cyclin D1, which is an important regulator of G1 to S phase in a variety of cancers, including breast cancer, to promote cell cycle progression [32–34]. Glycogen Synthase Kinase-3 (GSK-3), a serine/threonine protein kinase, was initially considered to be a key enzyme involved in glycogen metabolism [35], but is now recognized as a regulator of diverse cellular functions [36, 37]. Due to its kinase activity, GSK-3β is able to target cyclin D1 and β-catenin [38] [28] for ubiquitin-dependent proteasomal degradation. Our current study has shown that PSAT1 enhanced the stability of cyclin D1 via the induction of the phosphorylation of GSK-3β. GSK-3β was inactivated by phosphorylation, which resulted in its accumulation and the nuclear translocation of β-catenin [39, 40]. Consistently, we found that PSAT1 promoted the stability of β-catenin and its translocation into the nucleus through an enhancement of the phosphorylation of GSK-3β. β-catenin signaling has often been demonstrated to up-regulate the transcription of the cyclin-D1 protein [41]. It is worthy to note that our current study of PSAT1 focused on GSK-3β, through which PSAT1 eventually enhanced the proliferation and metastasis of tumor cells [8, 11]. Our current study found that PSAT1 enhanced the migration and invasiveness of ER-negative cells but reduced apoptosis (Additional file 2: Figure S1A and B). Given that previous studies have shown that GSK-3β is a promising target for cancer treatment, further research on the mechanism of PSAT1 and GSK-3β in ER-negative breast cancer may provide more valuable insight into optimal treatments for this type of breast cancer.

Yan et al. [42] reported that PSAT1 was a direct target of miR-340 and that its overexpression partially reversed miR-340-mediated inhibition of viability, invasion and EMT in ESCC cells. ATF4 transcriptionally activates serine biosynthetic genes in response to serine starvation in non-small cell lung cancer, and additionally, it has been shown to play a crucial role in the regulation of PSAT1 after OSN (Oct4, Sox2, and Nanog) was expressed in mouse embryonic stem cells [43, 44]. In this study, we found that the knockdown of ATF4 led to the down-regulation of PSAT1, and ChIP confirmed that ATF4 was bound to the ATF4-binding consensus sequences on the PSAT1 promoter in ER-negative breast cancer cells.

## Conclusions

To conclude, for the first time, our study demonstrated that PSAT1 was significantly up-regulated in ER-negative breast cancer. Consequently, this up-regulation was able to enhance the proliferation of ER-negative breast cancer cells in vitro via the GSK3β/β-catenin/cyclin D1 pathway and was able to promote tumor development in vivo. In addition, further investigation showed that PSAT1 was activated directly by ATF4 in ER-negative breast cancer. These results indicate that, as an oncogene, PSAT1 plays a vital role in the development of ER-negative breast cancer.

## Additional files

Additional files 1: Patients’ clinicopathological characteristics. (XLSX 58 kb)

Additional files 2: Figure S1. Effects of PSAT1 on the migration, invasion and apoptosis of ER-negative breast cancer cells. (A) Transwell assays were used to investigate changes in cell migration and invasion. **P < 0.01, ***P < 0.001, ****P < 0.0001. (B) Apoptosis assay based on flow cytometry shows that the suppression of PSAT1 increased the proportion of early apoptotic cells. (PDF 84 kb)

## Abbreviations

ATF4: Activating transcription factor 4
CCK-8: Cell Counting Kit-8
ChIP: Chromatin immunoprecipitation
ER: Estrogen receptor
ESCC: Esophageal squamous cell carcinoma
GSK3β: Glycogen Synthase Kinase-3β
IHC: Immunohistochemistry
NCCN: National Comprehensive Cancer Network
NSCLC: Non-small cell lung cancer
OSN: Oct4, Sox2, and Nanog
PHGDH: Phosphoglycerate dehydrogenase
pPYR: Phosphohydroxypyruvate
PSAT1: Phosphoserine aminotransferase 1
PSPH: 1-3-phosphoserine phosphatase
qRT-PCR: Quantitative real-time PCR
TCGA: The Cancer Genome Atlas
TMA: Tissue microarray
TNM: Tumor-Node-Metastasis
