# Supplementary figures and images for: PSAT1 is regulated by ATF4 and enhances cell proliferation via the GSK3β/β-catenin/cyclin D1 signaling pathway in ER-negative breast cancer
Source: J Exp Clin Cancer Res. 2017 Dec 8;36:179. doi: 10.1186/s13046-017-0648-4 (PMC5721480; doi:10.1186/s13046-017-0648-4)

**A**

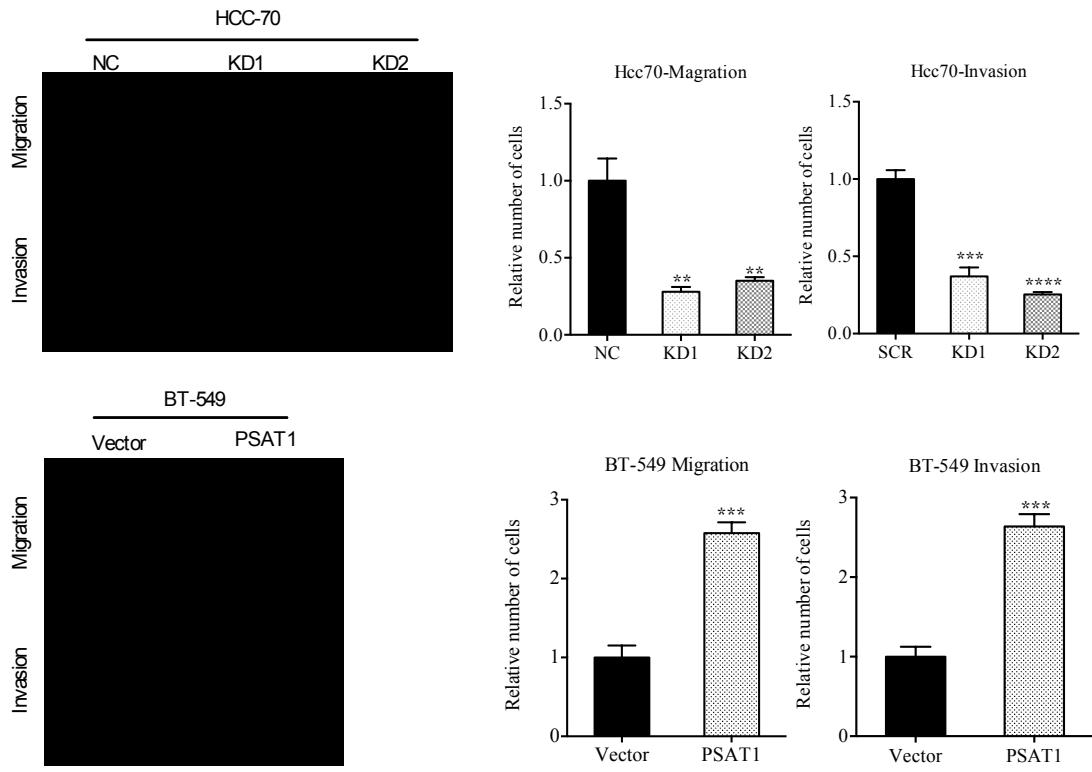

**B**

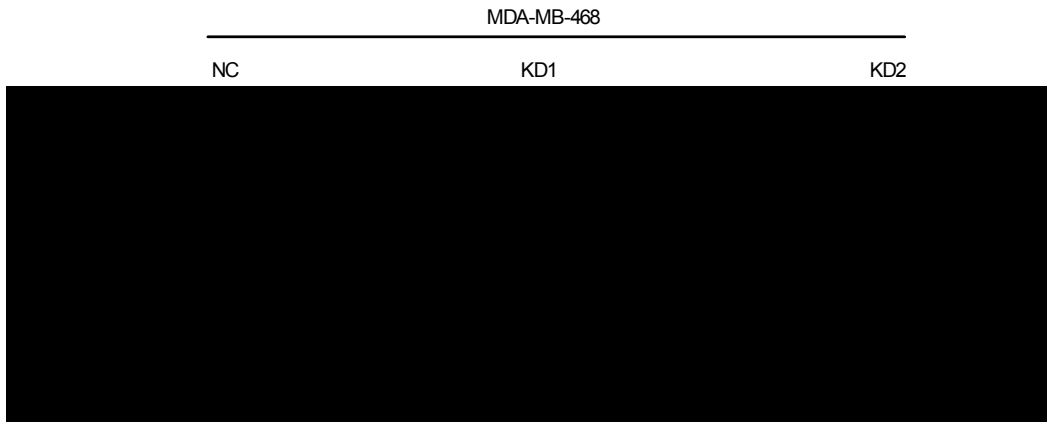

Supplement: Supplementary file 2 — Effects of PSAT1 on the migration, invasion and apoptosis of ER-negative breast cancer cells. (A) Transwell assays were used to investigate changes in cell migration and invasion. **P < 0.01, ***P < 0.001, ****P < 0.0001. (B) Apoptosis assay based on flow cytometry shows that the suppression of PSAT1 increased the proportion of early apoptotic cells. (PDF 84 kb) [file 13046_2017_648_MOESM2_ESM.pdf]
